# A multi-SNP association test for complex diseases incorporating an optimal P-value threshold algorithm in nuclear families

**DOI:** 10.1186/s12864-015-1620-3

**Published:** 2015-05-15

**Authors:** Yi-Ting Wang, Pei-Yuan Sung, Peng-Lin Lin, Ya-Wen Yu, Ren-Hua Chung

**Affiliations:** Institute of Statistics, National Tsing Hua University, Hsin-Chu, Taiwan; Department of Medical Science, National Tsing Hua University, Hsin-Chu, Taiwan; Division of Biostatistics and Bioinformatics, Institute of Population Health Sciences, National Health Research Institutes, Zhunan, Taiwan

## Abstract

**Background:**

Genome-wide association studies (GWAS) have become a common approach to identifying single nucleotide polymorphisms (SNPs) associated with complex diseases. As complex diseases are caused by the joint effects of multiple genes, while the effect of individual gene or SNP is modest, a method considering the joint effects of multiple SNPs can be more powerful than testing individual SNPs. The multi-SNP analysis aims to test association based on a SNP set, usually defined based on biological knowledge such as gene or pathway, which may contain only a portion of SNPs with effects on the disease. Therefore, a challenge for the multi-SNP analysis is how to effectively select a subset of SNPs with promising association signals from the SNP set.

**Results:**

We developed the Optimal P-value Threshold Pedigree Disequilibrium Test (OPTPDT). The OPTPDT uses general nuclear families. A variable p-value threshold algorithm is used to determine an optimal p-value threshold for selecting a subset of SNPs. A permutation procedure is used to assess the significance of the test. We used simulations to verify that the OPTPDT has correct type I error rates. Our power studies showed that the OPTPDT can be more powerful than the set-based test in PLINK, the multi-SNP FBAT test, and the p-value based test GATES. We applied the OPTPDT to a family-based autism GWAS dataset for gene-based association analysis and identified MACROD2-AS1 with genome-wide significance (*p*-value= 2.5 × 10^− 6^).

**Conclusions:**

Our simulation results suggested that the OPTPDT is a valid and powerful test. The OPTPDT will be helpful for gene-based or pathway association analysis. The method is ideal for the secondary analysis of existing GWAS datasets, which may identify a set of SNPs with joint effects on the disease.

**Electronic supplementary material:**

The online version of this article (doi:10.1186/s12864-015-1620-3) contains supplementary material, which is available to authorized users.

## Background

Genome-wide association studies (GWAS) have become a common approach to identifying single nucleotide polymorphisms (SNPs) associated with complex diseases. Traditional GWAS analysis tested individual SNPs associated with the disease. However, the significant SNPs only explained a small portion of heritability of the complex traits [1]. Complex diseases, such as hypertension, diabetes, and Alzheimer disease, are caused by the joint effects of multiple genes, while the effects of individual genes or SNPs are modest. The statistical power for identifying single SNPs with small effects can be low. Therefore, a method that considers the joint effects of multiple SNPs will be more powerful than the single-SNP test.

There have been many multi-SNP association tests proposed for genetic studies. Han and Pan [2] classified the multi-SNP tests for unrelated cases and controls into five categories. Methods in the first three categories compare the difference in allele frequencies [3, 4], Hardy-Weinberg disequilibrium [5], and linkage disequilibrium (LD) [6] between cases and controls. Methods in the other two categories are based on the genomic distance-based regression [7] and haplotype similarity approaches [8, 9]. Moreover, haplotype-based tests [10], which compare the difference in haplotype frequencies between cases and controls, can be classified in the same category of methods that compare the difference in allele frequencies.

Many family-based multi-SNP association tests are also available. A multi-SNP test [11] similar to the Hotelling-T^2^ test based on the single-SNP FBAT statistics [12] and a multi-SNP statistic [13] based on combining the weighted single-SNP FBAT statistics were developed. These methods can be classified into the category of comparing allele frequencies between affected siblings and controls (such as parents and unaffected siblings). The LD-based approach was also extended to a family-based method by comparing LD patterns between affected siblings and controls [14]. The transmission disequilibrium test (TDT) [15] was extended to multi-SNP tests based on the haplotype similarity approaches [16, 17]. Finally, several haplotype-based tests were also developed for analyzing family data [18–20].

Biological functions, such as genes or pathways, are commonly used to define SNP sets in the multi-SNP analysis. The multi-SNP analyses which select SNPs based on genes and pathways are referred to as the gene-based and pathway-based analyses, respectively. Current gene-based or pathway-based methods can be divided into three categories based on the data they use: case–control [21, 22], family [11, 23], and p-value based methods [24, 25]. Case–control and family-based methods use raw genotypes in unrelated cases and controls and families, respectively, while the p-value based methods use p-values from single-SNP association tests. One of the advantages for methods using raw genotypes is that permutation can be used to account for LD between SNPs and to correct for gene or pathway sizes. In contrast, p-value based methods have the advantages of accommodating different study designs, and p-values are easier to share in a consortium than the raw genotypes [26].

Gene or pathway-based methods provide biologically meaningful ways to select a set of SNPs within a gene or a pathway for the multi-SNP analysis. However, testing all the SNPs in the set may decrease the statistical power, particularly when there is only a portion of the SNPs that have effects on the disease in a large gene or a large pathway. Tag SNPs, which can predict the genotypes at other SNPs that are in LD with the tag SNPs, were used to select a representative subset of SNPs in a multimarker test [11] (implemented in the FBAT package). The multimarker test was shown to have similar power with the Bonferroni-Holm [27] method that controls family-wise error rate (FWER). A truncated product method [28] (referred to as the threshold method) was proposed to select SNPs with single-SNP p-values less than the pre-specified threshold in the set, and to test association only on the selected SNPs. A permutation or a Monte-Carlo simulation approach was used to account for LD between the SNPs and to obtain the p-value for the test. This method was more powerful than the methods that combined p-values for all of the SNPs in the set, such as Fisher’s method for combining p-values, and also more powerful than the method that controls FWER, such as Simes test [29]. A similar approach to the truncated product method was implemented in PLINK [30] (with the --set-test option), which used case–control or family genotype data in the analysis.

While the threshold method provides a powerful approach to the multi-SNP analysis, a p-value threshold needs to be specified before the analysis can be performed. In practice, the p-value threshold is usually specified as 0.05 (the default value in PLINK), even though this threshold may not be optimal. For example, if most of the causal SNPs have p-values less than 0.01, using a p-value threshold of 0.01 can result in higher power than using a p-value threshold of 0.05. The PLINK test can only use families with two parents and one affected children (triad) or families with one affected and one unaffected sib (discordant sib pair). However, general nuclear families with multiple siblings were sampled in many family-based studies [31–33] and the PLINK test could not adequately analyze these studies. Thus, a multi-SNP test that uses general nuclear families is essential.

We developed the optimal p-value threshold pedigree disequilibrium test (OPTPDT) to accommodate general nuclear families without a pre-specified p-value threshold. The OPTPDT uses a variable threshold algorithm to select SNPs with the strongest association signal. The OPTPDT method is based on the Pedigree Disequilibrium Test (PDT) [34], which can use general nuclear family structures. The method is not restricted to a single gene analysis, but can be applied to a gene-set or pathway analysis. We used simulations to demonstrate that the OPTPDT test has correct type I error rates under different scenarios. Further, we compared the power for the OPTPDT test with the set-based test in PLINK, the multi-SNP FBAT test, and the p-value based test GATES [25], under different scenarios. Finally, we applied the OPTPDT to the Autism Genome Project (AGP) family GWAS dataset for gene-based association analysis, and identified the MACROD2-AS1 gene with genome-wide significance for autism.

## Methods

### Pedigree disequilibrium test (PDT)

The OPTPDT was developed based on the PDT statistic. Therefore, we first review the PDT statistic. Two types of families, including the informative nuclear families and the informative discordant sibships, are considered in the PDT. At a SNP, an informative nuclear family consists of at least one triad, and each triad has one affected child as well as two genotyped parents, where one or both parents are heterozygous. An informative discordant sibship has at least one discordant sib pair (DSP), and each DSP has one affected as well as one unaffected sibling with different genotypes at the SNP. Here we consider families that contain an informative nuclear family and/or an informative discordant sibship.

Consider two alleles, *A*_1_ and *A*_2_, at the SNP. For an affected child, there is a pair of alleles transmitted and not transmitted to the child from a parent. Define random variables ${X}_{T_i}$ and ${X}_{S_j}$ for a triad *i* and a DSP *j*, respectively, as:

$$ {X}_{T_i}=\left(\mathrm{number}\ \mathrm{of}\ {A}_1\ \mathrm{transmitted}\right) - \left(\mathrm{number}\ \mathrm{of}\ {A}_1\ \mathrm{not}\ \mathrm{transmitted}\right) $$

$$ {X}_{Sj}=\left(\mathrm{number}\ \mathrm{of}\ {A}_1\ \mathrm{i}\mathrm{n}\ \mathrm{affected}\ \mathrm{s}\mathrm{i}\mathrm{b}\right) - \left(\mathrm{number}\ \mathrm{of}\ {A}_1\ \mathrm{i}\mathrm{n}\ \mathrm{unaffected}\ \mathrm{s}\mathrm{i}\mathrm{b}\right) $$

within a nuclear family.

Let *n*_*T*_ be the number of triads and *n*_*S*_ be the number of DSPs in the nuclear family. Then *X*_*T*_ and *X*_*S*_ are defined as ${X}_T={\displaystyle {\sum}_{i=1}^{n_T}}{X}_{T_i}$ and ${X}_S={\displaystyle {\sum}_{j=1}^{n_S}}{X}_{S_j}$, respectively. The PDT statistic for the nuclear family was previously described by [34] and is defined as $D=\frac{1}{n_T+{n}_S}\left({X}_T+{X}_S\right)$. If there are *N* nuclear families, then the PDT statistic for the SNP is defined as:

1 $$ {T}^2=\frac{{\left({\displaystyle {\sum}_{i=1}^N}{D}_i\right)}^2}{{\displaystyle {\sum}_{i=1}^N}{D_i}^2} $$

Under the null hypothesis of no linkage or no association, *T*^2^ is asymptotically chi-square, with 1 degree of freedom. The statistic takes into consideration minor alleles with risk or protective effects since the statistic takes a squared value.

### Optimal threshold pedigree disequilibrium test (OPTPDT)

Assume there are *n* SNPs in a pre-defined chromosomal region. The region can be defined by the biological functions, such as introns, exons, genes, or pathways. Thus, our method is not restricted to a single gene analysis, but can be applied to a gene-set or pathway analysis. The null hypothesis for the test is that none of the SNPs in the region are associated with the disease. For each SNP in the region, the PDT statistic and its corresponding p-value is calculated. We define four variable p-value thresholds (i.e. 0.05, 0.03, 0.01, and 0.005), and SNPs with p-values less than each of the thresholds are selected. For a p-value threshold *k*, let ***L***_*k*_ be a vector containing the PDT statistics with p-values < *k*. The test statistic *Y*_*k*_ for threshold *k* is defined as the sum of all the PDT statistics in ***L***_*k*_. If no PDT statistics have p-values less than threshold *k*, ***L***_*k*_ is an empty set and *Y*_*k*_ is not calculated.

Our goal is to select an optimal threshold *k* so that ***L***_*k*_ contains the SNPs with the most significant association signal over the four possible values of *k*. However, each *Y*_*k*_ is not directly comparable because *Y*_*k*_ has a different distribution for each *k*. Therefore, we standardize *Y*_*k*_ to a Z-score, which is calculated as ${Z}_k=\frac{Y_k-{\mu}_k}{\sigma_k}$, where *μ*_*k*_ and *σ*_*k*_ are the mean and standard deviation for *Y*_*k*_ under the null. We use a permutation procedure to generate the statistics under the null for *Y*_*k*_ and estimate *μ*_*k*_ and *σ*_*k*_ based on the null statistics. For a permutation, we randomly permute the transmitted and non-transmitted alleles from parents to all children simultaneously for each family, and the PDT statistic for each family is calculated. We perform the permutations for *m* times, and each permutation *j* results in a permuted statistic ${Y}_{k_j}$. Then the estimates for *μ*_*k*_ and *σ*_*k*_ are calculated as $\widehat{\mu_k}={\displaystyle {\sum}_{j=1}^m}{Y}_{k_j}/m$ and $\widehat{\sigma_k}=\sqrt{{\displaystyle {\sum}_{j=1}^m}{\left({Y}_{k_j}-\widehat{\mu_k}\right)}^2/\left(m-1\right)}$, respectively. The statistics *Y*_*k*_ and ${Y}_{k_j}$ are standardized as *Z*_*k*_ and ${Z}_{k_j}$, respectively, based on $\widehat{\mu_k}$ and $\widehat{\sigma_k}$ for each *k*. Finally, we define the OPTPDT statistic *M* as max(*Z*_0.05_, *Z*_0.03_, *Z*_0.01_, *Z*_0.005_) and for each permutation *j*, ${M}_j= \max \left({Z}_{0.05_j},{Z}_{0.03_j},{Z}_{0.01_j},{Z}_{0.005_j}\right)$. The p-value for the OPTPDT is calculated as the number of *M*_*j*_ ≥ *M* divided by *m*.

The OPTPDT algorithm is summarized as follows.

1. For each SNP in the region, calculate the PDT statistic, *T*^2^, and its corresponding p-value.
2. Assume the variable p-value thresholds are 0.05, 0.03, 0.01, and 0.005. Select the SNPs with p-values less than each of the thresholds. For threshold *k*, ${\boldsymbol{L}}_k=\left\{{T_1}^2,{T_2}^2,\dots, {T_{n_k}}^2\right\}$, where *T*_*i*_^2^ is the PDT statistic *T*^2^ for SNP *i* with a p-value < *k*, and *n*_*k*_ is the number of SNPs in ***L***_*k*_ with p-values < *k* .
3. For each ***L***_*k*_, calculate ${Y}_k={\displaystyle {\sum}_{i=1}^{n_k}}{T_i}^2$.
4. Perform the permutation procedure for *m* times. For each permutation *j*, repeat steps 2–3, and obtain ${Y}_{k_j}$.
5. Standardize the statistics *Y*_*k*_ and ${Y}_{k_j}$ in each permutation based on the *m* permuted statistics, and obtain *Z*_*k*_ and ${Z}_{k_j}$ for each *k*.
6. Select *M* = max (*Z*_0.05_, *Z*_0.03_, *Z*_0.01_, *Z*_0.005_). For each permutation *j*, select *Mj* = max (*Z*_0.05 j_ Z _0.03 j_ Z_ 0.01 j_ Z_ 0.005 j_
7. The p-value is calculated as $\frac{\mathrm{number}\ \mathrm{of}\ \left({M}_j\ge M\right)}{m}$.

When the transmitted and non-transmitted alleles are permuted, the permutation simply results in a sign change in the PDT statistic. That is,

2 $$ D=\frac{1}{n_T+{n}_S}\left({X}_T+{X}_S\right)=-\frac{1}{n_T+{n}_S}\left({X}_{(T)}+{X}_{(S)}\right) $$

where *X*_(*T*)_ and *X*_(*S*)_ are the permuted statistics. The argument is still true when there are different numbers of affected and unaffected siblings. This property allows us to perform permutations even when parents are missing by simply permuting the sign of the PDT statistic for the family. Similarly, if we permute the transmitted and non-transmitted haplotypes at multiple SNPs, the permutations simply result in sign changes in the statistics *D* for all of the SNPs. Thus, we permute the signs of the PDT statistics for all SNPs simultaneously so that LD among SNPs can be maintained. These important properties of the algorithm are demonstrated in detail in Additional file 1. Also note that linkage is maintained by permuting the transmitted and non-transmitted alleles from parents to all children simultaneously in a family, as the identity-by-descent (IBD) status between children is not affected by the permutation.

### Simulations

We used simulation studies to evaluate the type I error and the power of the OPTPDT. Our simulation procedures occurred in two steps. In the first step, we used HAPGEN version 2 [35] to simulate haplotypes with allele frequencies and LD structures that were similar to the data collected from the European population in the HapMap Project [36]. We randomly selected 10 genes on chromosome 1 for the simulated regions. A total of 10,000 haplotypes in the 10 genes were simulated. In the second step, SeqSIMLA [37] was used to simulate nuclear families based on the 10,000 haplotypes. SeqSIMLA performed random mating and gene dropping based on the 10,000 haplotypes to generate pedigrees. We used the prevalence model in SeqSIMLA to simulate the disease status. In the prevalence model, the odds ratios for the disease loci and disease prevalence were specified. A logistic penetrance function described as follows was used to determine the affection status.

$\mathrm{P}\left(\mathrm{Affected}\ \Big|X\right) = \frac{ \exp \left(\upalpha + \boldsymbol{B}\boldsymbol{X}\right)}{1+ \exp \left(\upalpha + \boldsymbol{B}\boldsymbol{X}\right)}$, where ***X*** is a vector of genotype coding based on the additive, dominant or recessive disease model, for the *n* disease SNPs *X*_1_, …, *X*_*n*_; α is used to determine the disease prevalence; ***B*** = (*β*_1_, …, *β*_*n*_) represents the effect sizes for the disease SNPs. The disease prevalence was specified as 5 %.

We simulated different sample sizes (500 and 1000 families), family structures (nuclear family, triad, and discordant sibship), and different numbers of genes (1 and 10 genes). In a nuclear family, there were two parents and three siblings, which had one affected sibling and two unaffected siblings. A triad consisted of two parents and one affected sibling. A discordant sibship had two missing parents and one affected sibling and two unaffected siblings. There were 46 SNPs in 1 gene and 1207 SNPs in the 10 genes. For the type I error simulations, none of the SNPs in the region were associated with the disease. For the power simulations, we simulated different odds ratios (1.1, 1.2, and 1.3) for the disease SNPs, different disease models (recessive, additive, and dominant), and different numbers of disease SNPs (5 and 10). The odds ratios for all disease SNPs were assumed to be the same. The disease SNPs were randomly selected from the SNPs that were not in LD, with minor allele frequencies (MAF) > 1 %. The MAF for the 5 disease SNPs were 0.1, 0.13, 0.2, 0.4, and 0.42, while the MAF for the 10 disease SNPs were 0.04, 0.05, 0.1, 0.1, 0.13, 0.2, 0.37, 0.4, 0.42, and 0.43.

We compared the power of the OPTPDT with two other family-based multi-SNP tests, PLINK and FBAT [11], and a p-value based method, GATES [25], under different scenarios. PLINK is also a threshold method that uses a default p-value threshold (i.e., 0.05). The FBAT uses all of the SNPs or a pre-selected tag SNPs in the set for a multivariate test. GATES uses an extended Simes procedure to calculate an overall p-value for a set of p-values obtained from single-SNP association tests. The PDT was used to calculate the single-SNP association p-values for GATES. Because PLINK can only use triads or DSPs, we simulated triads for the power comparison. Tag SNPs, selected based on the “LD based SNP pruning” function in PLINK, in a simulated region were used for FBAT.

### Real dataset analysis

We applied the OPTPDT to a GWAS dataset from the Autism Genome Project (AGP) [38–40]. The dataset containing the genotype and phenotype information was downloaded from dbGaP (accession phs000267.v4.p2). Samples in the data were recruited from North America and Europe. The dataset consisted of nuclear families collected from two stages. The stage 1 data contained about 1400 autism spectrum disorder (ASD) families genotyped on the Illumina Infinium 1 M-single SNP array, while the stage 2 data consisted of 1301 ASD families genotyped on either the Illumina Infinium 1 M-single or the Illumina 1 M-duo array. The combined data from both stages were used for the analysis. Strict autism as defined in the phenotype file provided by the Project was used for the phenotype in the analysis. Written informed consent for participation in the AGP study was obtained from all participants and research in the AGP study was approved by institutional review boards from all institutions involved in the AGP study [39]. The analysis in the present study was approved by the Institutional Review Board (IRB) of the National Health Research Institutes in Taiwan (IRB protocol # EC1020503-E). The same quality control (QC) procedures as described in [38] were applied to the combined data. Families clustered with the European samples in the HapMap project based on the principal component analysis performed using the SNPRelate package [41] were extracted. After QC, our analysis dataset consisted of 1192 families with 1206 children diagnosed with strict autism and 2384 unaffected individuals and 822,668 SNPs. We downloaded the hg18 gene annotations from the UCSC genome browser website [42]. SNPs within each gene were defined as a test set. There were 17,016 genes used in the analysis, and a total of 368,584 SNPs were mapped to the genes.

## Results and discussion

### Type I error and power

**Fig. 1:**
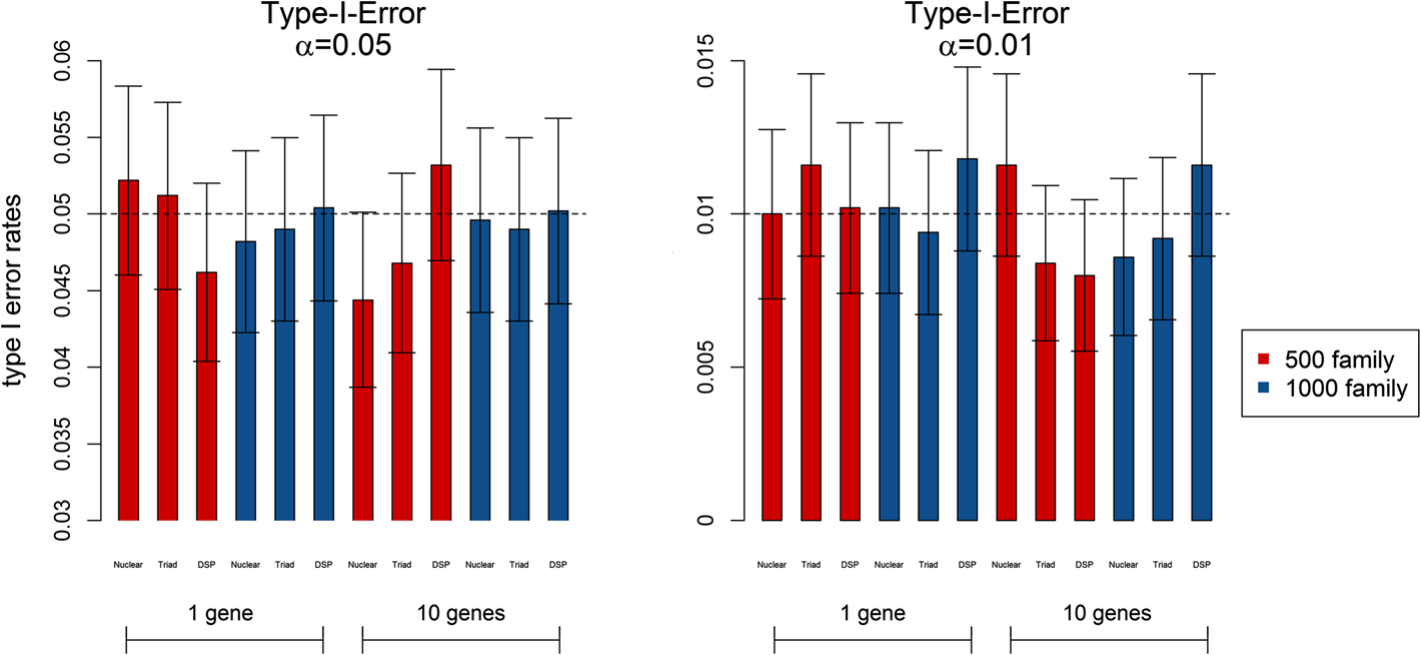
Type I error rates under different scenarios at *α* = 0.05 and 0.01. The *error bars* represent the 95 % confidence intervals for the type I error rates

Figure 1 shows the type I error rates under different scenarios (such as different family structures, numbers of families, and numbers of genes) at the significance levels (α) of 0.05 and 0.01. The type I error rates for the OPTPDT are close to the nominal levels, and all of the 95 % confidence intervals contain the expected levels.

**Fig. 2:**
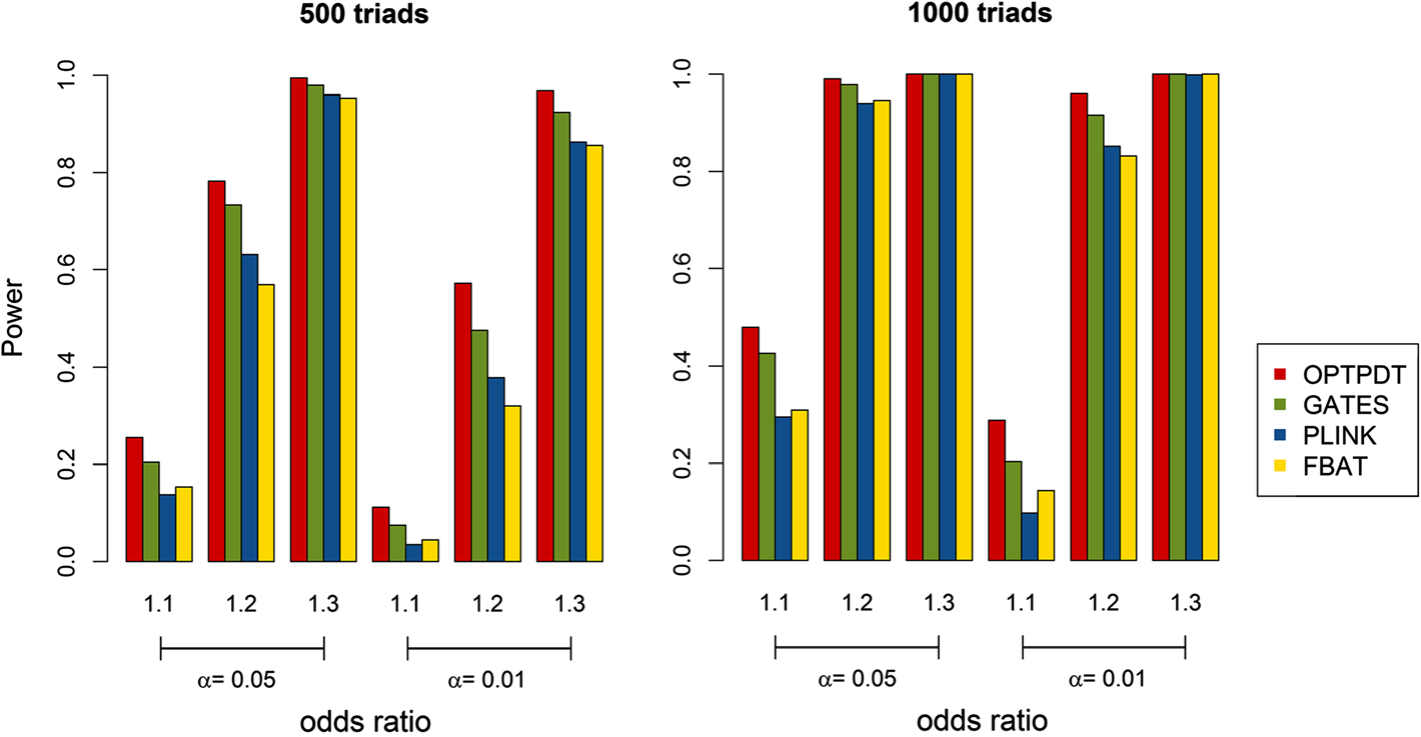
Power comparison for different odds ratios (1.1, 1.2, and 1.3) and different numbers of triads (500 and 1000) at the significance levels (α) of 0.05 and 0.01

**Fig. 3:**
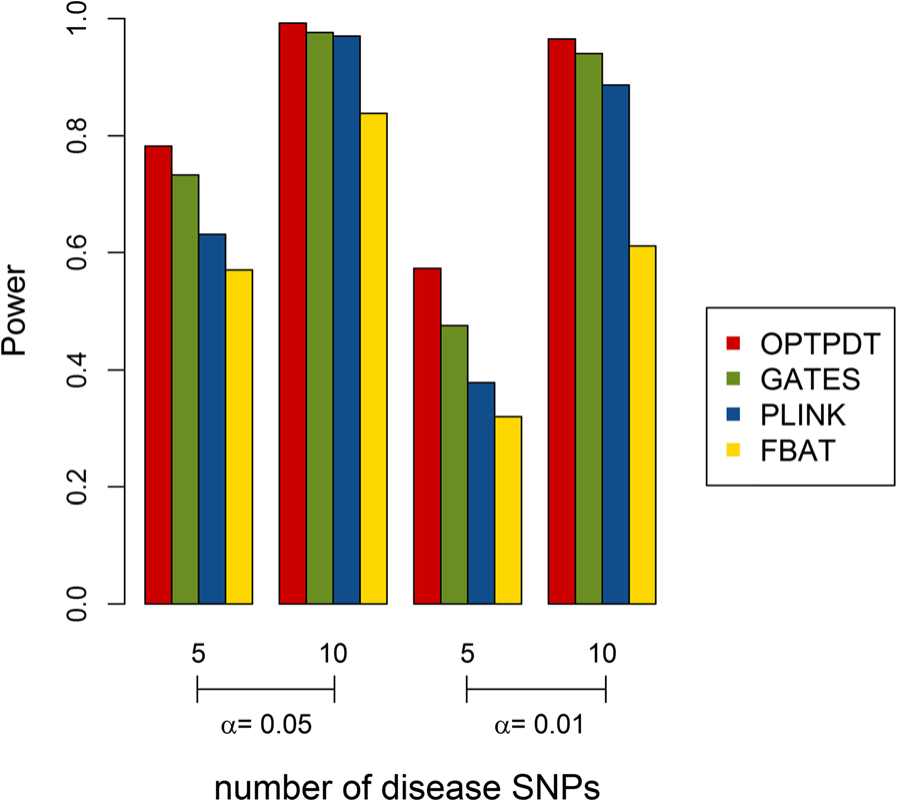
Power comparison for different numbers of disease SNPs (5 and 10) at the significance levels (α) of 0.05 and 0.01

We compared the power for the OPTPDT test with PLINK, FBAT and GATES under different scenarios. The default parameter setting was 500 triads, 5 disease SNPs with the odds ratios of 1.2, an additive model, and 1 gene for testing. Parameters were changed one or two at a time for each simulation scenario. Figure 2 shows the power comparison when the disease SNPs have different odds ratios of (1.1, 1.2, and 1.3) based on 500 and 1000 triads, respectively. The power for the OPTPDT is higher than PLINK, FBAT, and GATES with different odds ratios for either 500 or 1000 triads. As expected, the power for the four tests increased when the odds ratios increased for the disease SNPs. Figure 3 shows the power comparison with 5 and 10 disease SNPs. A similar power pattern was observed that the OPTPDT has the highest power compared to PLINK, FBAT, and GATES with 5 or 10 disease SNPs. The power for all the tests increased when more disease SNPs were simulated.

**Fig. 4:**
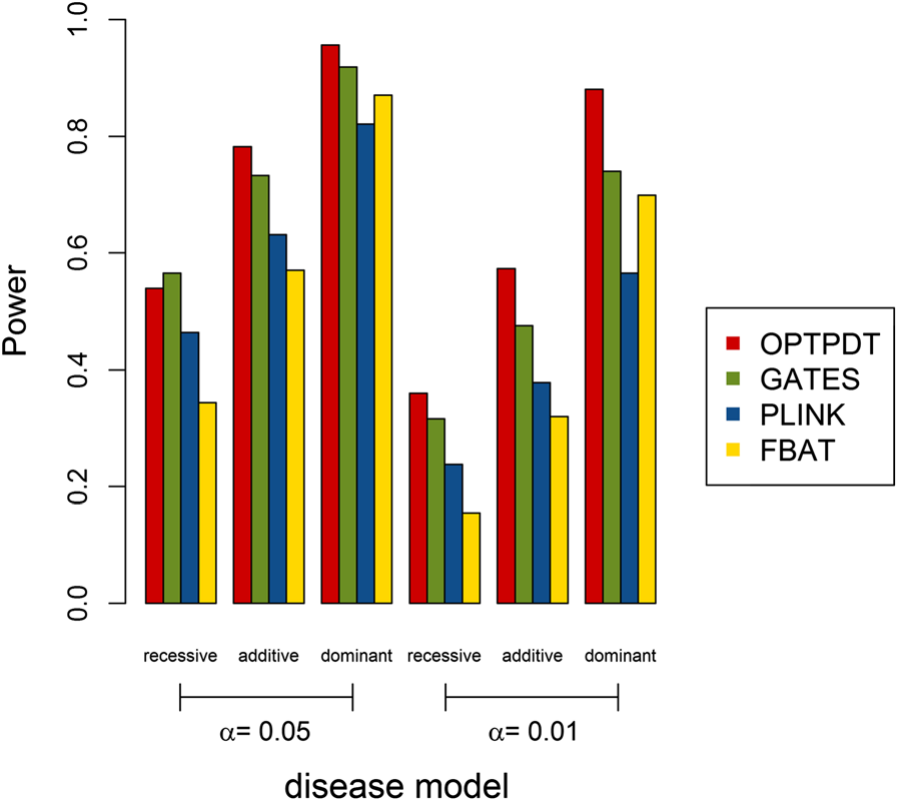
Power comparison under different disease models (recessive, additive, and dominant) at the significance levels (α) of 0.05 and 0.01

Figure 4 shows the power comparison in the recessive, additive, and dominant models. Again, the OPTPDT maintains the highest power under different disease models, except that GATES has slightly higher power than the OPTPDT under the recessive model at the 0.05 significance level. As seen in Figs 1, 2, 3 and 4, FBAT has the lowest power compared to the OPTPDT, PLINK, and GATES in many of the scenarios. Moreover, interestingly, GATES, a p-value based test, is more powerful than PLINK and FBAT, which use raw genotypes, in these simulation scenarios.

**Fig. 5:**
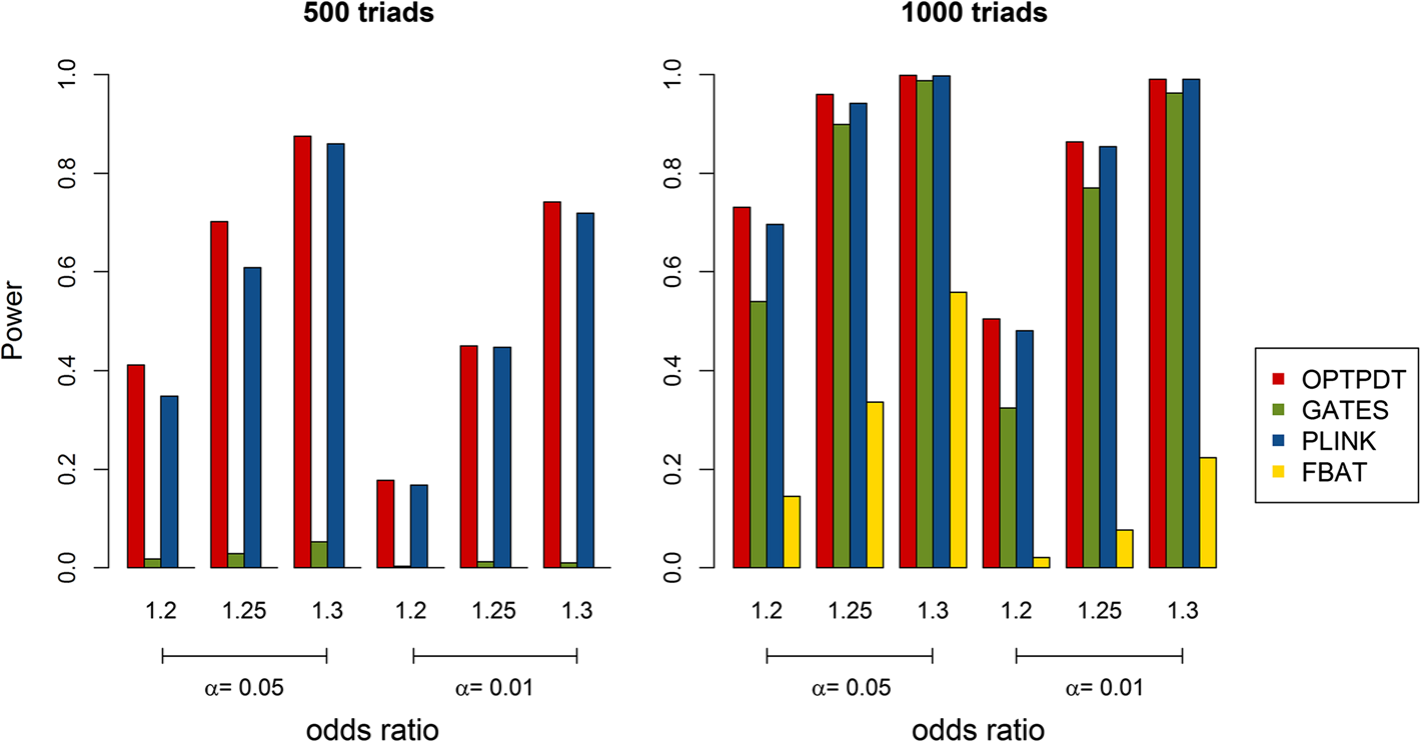
Power comparison with different odds ratios (1.2, 1.25, and 1.3) for 10 disease SNPs at 10 genes at the significance levels (α) of 0.05 and 0.01

Figure 5 shows the power comparison for a region that has 10 genes. A total of 10 disease SNPs were simulated with the same odds ratios of 1.2, 1.25 and 1.3. Similar to the observations for 1 gene, the OPTPDT has higher power than PLINK, FBAT, and GATES in all of the scenarios. FBAT has no power with 500 families, due to the large degrees of freedom for the multivariate test, and GATES also has relatively low power with 500 families, compared to the OPTPDT and PLINK. These results suggest that there is an advantage to selecting a promising subset of SNPs for analysis, particularly when the proportion of causal SNPs is small in the set of SNPs that are analyzed. We also randomly selected another 5 and 10 disease SNPs that were not in LD with MAF > 1 % for the power simulations, and found similar power patterns (Data not shown). Therefore, our power results represent a general power pattern for testing the joint effects of SNPs with MAF > 1 % for different methods compared in this study given the simulation settings.

### AGP analysis

**Fig. 6:**
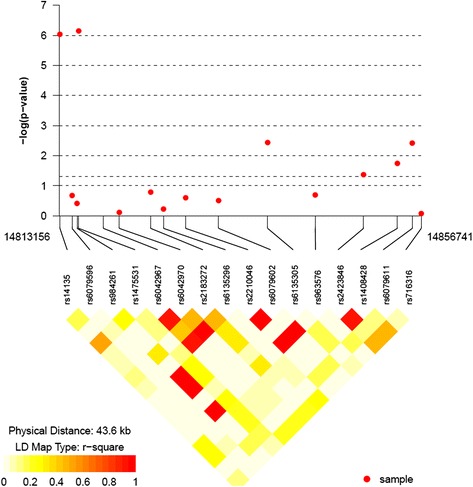
The association p-values for the individual SNPs and their LD structures in MACROD2-AS1

We applied the OPTPDT to the AGP GWAS dataset. The 10 most significant genes based on the OPTPDT p-values are shown in Table 1. The p-value for the most significant gene, MACRO domain containing 2 antisense RNA 1 (MACROD2-AS1), passed the multiple testing threshold of 2.93 × 10^− 6^ for testing 17,016 genes. We show the association p-values for the individual SNPs and their LD structures in MACROD2-AS1 in Fig. 6. As seen in the Figure, two SNPs, rs14135 and rs1475531, in the gene show highly significant marginal p-values of 9.33 × 10^− 7^ and 7.08 × 10^− 7^, respectively, while other SNPs, such as rs6135305 with p-value of 3.63 × 10^− 3^, rs2423846 with p-value of 4.27 × 10^− 2^, rs1408428 with p-value of 1.77 × 10^− 2^, and rs6079611 with p-value of 3.83 × 10^− 3^, also show some marginal significance. However, none of the p-values for the SNPs in the gene would pass the commonly used genome-wide multiple-testing threshold (i.e., 5 × 10^− 8^) for individual SNP analysis. We also show the GATES, PLINK and FBAT p-values for the 10 genes. The GATES, PLINK and FBAT test p-values were also significant (p-value < 0.05) for most of the 10 genes.

**Table 1:** The 10 most significant genes in the AGP analysis for autism identified by the OPTPDT

			*P*-value			
Chromosome	Gene	Number of SNPs	OPTPDT	GATES	PLINK	FBAT
20	MACROD2-AS1	16	2.5 × 10^− 6^	7.2 × 10^− 6^	1.5 × 10^− 3^	2.2 × 10^− 2^
1	CYMP	11	5.0 × 10^− 5^	2.8 × 10^− 4^	1.5 × 10^− 3^	3.9 × 10^− 4^
5	CAPSL	18	5.0 × 10^− 5^	1.2 × 10^− 3^	1.5 × 10^− 3^	1.1 × 10^− 2^
13	LINC00548	7	4.0 × 10^− 4^	6.2 × 10^− 3^	2.5 × 10^− 3^	5.3 × 10^− 3^
18	IMPA2	28	4.0 × 10^− 4^	5.4 × 10^− 3^	2.5 × 10^− 2^	1.5 × 10^− 1^
3	GMNC	2	4.0 × 10^− 4^	2.3 × 10^− 4^	2.0 × 10^− 4^	6.9 × 10^− 5^
11	LUZP2	206	5.0 × 10^− 4^	5.2 × 10^− 2^	3.0 × 10^− 1^	6.5 × 10^− 1^
17	KRTAP4-6	1	5.0 × 10^− 4^	1.3 × 10^− 3^	1.0 × 10^− 3^	1.7 × 10^− 3^
17	ERN1	16	5.0 × 10^− 4^	7.1 × 10^− 3^	3.5 × 10^− 3^	2.8 × 10^− 1^
2	IRS1	18	5.0 × 10^− 4^	1.4 × 10^− 3^	1.3 × 10^− 2^	4.7 × 10^− 3^

### Discussion

PLINK requires a user-specified p-value threshold while the OPTPDT automatically identifies an optimal threshold for selecting a subset of promising SNPs for the analysis. The OPTPDT uses four variable p-value thresholds (i.e. 0.05, 0.03, 0.01, and 0.005) to select SNPs. More thresholds can be incorporated in the OPTPDT for the SNP selection based on the same algorithm. However, increasing the number of thresholds will increase the computational complexity in the algorithm. Further, we evaluated the power for the OPTPDT which uses ten p-value thresholds (0.05, 0.04, 0.03, 0.02, 0.01, 0.009, 0.007, 0.005, 0.003, and 0.001) based on the default setting in the power simulations, and found that the power is similar to the power for the OPTPDT using the four thresholds (Data not shown). Therefore, when the four thresholds were used, the OPTPDT was still capable of identifying an optimal set of SNPs based on the simulations.

Although the permutation strategy of permuting the transmitted and non-transmitted alleles in families were used in other family-based association tests [43, 44], the property of the permutation procedure with the test statistic has not been explored in detail. We discussed the property of the permutation procedure in Additional file 1 in different situations, such as permutations at one SNP or multiple SNPs in LD, families with different numbers of affected and unaffected siblings, and recombination between SNPs. The general conclusion is that permuting the transmitted and non-transmitted haplotypes in a region from parents to all siblings results in simultaneous sign changes in single-SNP PDT statistics for all markers in the region. This important property can be applied to future family-based association tests incorporating permutations.

Currently, the OPTPDT uses nuclear families in the analysis. The method cannot be directly applied to the extended pedigrees, because permuting transmitted and non-transmitted alleles at a SNP in the extended pedigrees does not result in a sign change in the PDT statistic. Further, when there are missing data, permuting transmitted and non-transmitted alleles in the extended pedigrees is not straightforward. Moreover, the OPTPDT currently considers only dichotomous trait. The extended PDT which uses a quantitative trait [45] can be potentially incorporated in the OPTPDT algorithm. It is our future work to develop an efficient permutation strategy in the OPTPDT for extended pedigrees and quantitative traits.

The optimal p-value threshold algorithm can also be applied to other association test statistics, such as the FBAT statistic, test statistics based on linear and logistic regressions from unrelated subjects, or test statistic based on mixed-model from related subjects, as long as an appropriate permutation strategy is used. To be more specific, the single-marker PDT statistic in Step 1 of the OPTPDT algorithm can be replaced by another test statistic for association. For unrelated subjects, the permutation procedure in Step 4 of the OPTPDT algorithm can be performed by randomly permuting the trait values among subjects. For related subjects, the correlation structures among subjects should be considered in the permutation procedure.

The OPTPDT is designed to analyze only common variants (e.g., variants with MAF > 1 %), because the algorithm uses the single-SNP p-values for identifying an optimal subset of SNPs. Hence, the OPTPDT is suitable for analyzing GWAS data. For rare variants from sequencing studies, their single-SNP p-values may not be informative to the SNP selection in the OPTPDT. Therefore, other family-based methods designed for rare variants, such as methods proposed in [46, 47], should be used to test a set of rare variants.

Our AGP analysis identified a significant gene, MACROD2-AS1, associated with autism. Interestingly, analysis based on the stage 1 AGP data identified a genome-wide significant SNP, rs4141463, in the intron region of MACROD2, which is located downstream 1 MB of MACROD2-AS1 on chromosome 20, for autism. However, the role of MACROD2-AS1 in the function of MACROD2 is not clear based on our literature search. As several SNPs in MACROD2-AS1 show p-values < 0.05, we performed haplotype analysis for the 16 SNPs in the gene to investigate whether there are haplotype effects in the gene on autism, using the haplotype-based transmission disequilibrium test (hap-TDT) implemented in PLINK. The results are shown in Additional file 2. The haplotype analysis identified a common haplotype (GCGCCGGGAAGAGGAG) with frequency of 11 % that shows significant p-value of 1.0 × 10^− 3^ based on the multiple testing threshold of 3.8 × 10^− 3^ for testing 13 haplotypes.

We evaluated the performance of the OPTPDT in terms of run time on a Linux server with Xeon 2.0 GHz CPUs. The OPTPDT spent averages of 5 and 7 s for the set of 46 SNPs in one simulated replicate of 500 and 1000 triads, respectively, based on 2000 permutations. The OPTPDT spent averages of 1 min and 6 s and 2 min and 46 s for the set of 1207 SNPs in one simulated replicate of 500 and 1000 triads, respectively. For the AGP analysis, the OPTPDT spent about 5 h and 44 s analyzing 1770 genes on chromosome 1. The analyses for different chromosomes can be run in parallel as the analyses are independent. Therefore, the OPTPDT can finish analyzing a large GWAS dataset in a reasonable time frame.

## Conclusions

We developed the multi-SNP association test, OPTPDT, using a variable p-value threshold algorithm to select SNPs with the strongest association signal at a particular p-value threshold. We used simulations to verify that the OPTPDT had correct type I error rates. We also used simulations to compare the power of the OPTPDT with PLINK, FBAT, and GATES. The OPTPDT had the highest power in most of the scenarios, followed by GATES, PLINK and FBAT. The simulation results showed that FBAT had the lowest power in all of the simulation scenarios. These results demonstrate that the threshold methods (i.e., OPTPDT and PLINK) are more powerful than a method using all of the SNPs without selection, particularly when only a portion of the SNPs in the set are causal.

The OPTPDT can be helpful for gene-based or pathway association analysis. The method is ideal for the secondary analysis of existing GWAS datasets, which may identify a set of SNPs with joint effects on the disease. We have implemented the method into an efficient software package using C++, which can be downloaded for free from http://optpdt.sourceforge.net.

## Additional files

Additional file 1:
**Permutation property for the PDT statistic.**
Additional file 2:
**Haplotype analysis results for MACROD2-AS1.**

## Abbreviations

GWAS: Genome-wide association studies
SNPs: Single nucleotide polymorphisms
OPTPDT: Optimal P-value Threshold Pedigree Disequilibrium Test
LD: Linkage disequilibrium
TDT: Transmission disequilibrium test
FWER: Family-wise error rate
PDT: Pedigree disequilibrium test
AGP: Autism Genome Project
DSP: Discordant sib pair
IBD: Identity-by-descent
ASD: Autism spectrum disorder
QC: Quality control
MACROD2-AS1: MACRO domain containing 2 antisense RNA 1
