# Supplementary material for: A multi-SNP association test for complex diseases incorporating an optimal P-value threshold algorithm in nuclear families
Source: BMC Genomics. 2015 May 15;16(1):381. doi: 10.1186/s12864-015-1620-3 (PMC4433014; doi:10.1186/s12864-015-1620-3)
Supplement: Additional file 1: — Permutation property for the PDT statistic. [file 12864_2015_1620_MOESM1_ESM.docx]

We first discuss the permutation property for one SNP. Consider a nuclear family with two parents, *m* affected and *n* unaffected siblings.

Let $G_{T_{i}}$ be the number of allele 1 transmitted from parents, and let $G_{{NT}_{i}}$ be the number of non-transmitted allele 1 from parents, to affected sib *i* , where *i*=1,2,…,*m*

Let ${G^{'}}_{T_{j}}$ be the number of allele 1 transmitted from parents, and let ${G^{'}}_{{NT}_{j}}$ be the number of non-transmitted allele 1 from parents, to unaffected sib *j* , where *j*=1,2,…,*n*

Table A.1 shows the transmitted and non-transmitted statistics for the affected and unaffected sibs for the nuclear family.

Since the total number of allele 1 in parents is fixed, $G_{T_{1}}+G_{{NT}_{1}}=G_{T_{2}}+G_{{NT}_{2}}=\ldots=G_{T_{m}}+G_{{NT}_{m}}={G^{'}}_{T_{1}}+{G^{'}}_{{NT}_{1}}={G^{'}}_{T_{2}}+{G^{'}}_{{NT}_{2}}=\ldots={G^{'}}_{T_{n}}+{G^{'}}_{{NT}_{n}}$

If *m*=*n*

$$G_{T_{1}}-{G^{'}}_{T_{1}}={G^{'}}_{{NT}_{1}}-G_{{NT}_{1}}$$

$$G_{T_{2}}-{G^{'}}_{T_{2}}={G^{'}}_{{NT}_{2}}-G_{{NT}_{2}}$$

.

.

.

$$G_{T_{n}}-{G^{'}}_{T_{n}}={G^{'}}_{{NT}_{n}}-G_{{NT}_{n}}$$

$$\therefore\sum_{i=1}^{n} G_{T_{i}}-\sum_{j=1}^{n} {G^{'}}_{T_{j}}=\sum_{j=1}^{n} {G^{'}}_{{NT}_{j}}-\sum_{i=1}^{n} G_{{NT}_{i}} -----(1)$$

$$\sum_{i=1}^{m} G_{T_{i}}-\sum_{j=1}^{m} {G^{'}}_{T_{j}}=\sum_{j=1}^{m} {G^{'}}_{{NT}_{j}}-\sum_{i=1}^{m} G_{{NT}_{i}} -----(2)$$

We define $n_{T}$ = number of triads (two parents and one affected offspring) and

$n_{S}$ = number of discordant sib pair (one affected and one unaffected sibs) in the nuclear family.

Let $X_{T}$ be the sum of difference in allele counts between the transmitted and the non-transmitted alleles over all triads. Let $X_{S}$ be the sum of difference in allele counts between the affected and unaffected sibs over all discordant sib pairs.

$$Therefore , X_{T}=\sum_{i=1}^{m} (G_{T_{i}}-G_{{NT}_{i}})$$

$$\mathrm{If}m=n, X_{s}=\sum_{i=1}^{m} G_{T_{i}}-\sum_{j=1}^{n} {G^{'}}_{T_{j}}$$

$$\mathrm{If}m\neq n, X_{s}=n\sum_{i=1}^{m} G_{T_{i}}-m\sum_{j=1}^{n} {G^{'}}_{T_{j}}$$

The PDT statistic for this nuclear family is calculated as $\frac{1}{n_{T}+n_{S}}(X_{T}+X_{S})$

After permuting the transmitted and non-transmitted alleles, the statistics for $X_{T}$ and $X_{S}$ can be calculated as:

$$X_{(T)}=\sum_{i=1}^{m} (G_{{NT}_{i}}-G_{T_{i}})$$

$$X_{(S)}=\sum_{i=1}^{m} G_{{NT}_{i}}-\sum_{j=1}^{n} {G^{'}}_{{NT}_{j}}$$

We investigate the relationship between $X_{T}+X_{S}$ and $X_{(T)}+X_{\left( S \right)}$ in the following three cases.

**(Case 1) The numbers of affected and unaffected sibs are equal. (*m*=*n*)**

$$X_{T}=\sum_{i=1}^{m} (G_{T_{i}}-G_{{NT}_{i}})=-\sum_{i=1}^{m} \left( G_{{NT}_{i}}-G_{T_{i}} \right)=-X_{(T)}$$

$$X_{S}=\sum_{i=1}^{m} G_{T_{i}}-\sum_{j=1}^{n} {G^{'}}_{T_{j}}=\sum_{i=1}^{n} G_{T_{i}}-\sum_{j=1}^{n} {G^{'}}_{T_{j}}$$

$$X_{\left( S \right)}=\sum_{i=1}^{m} G_{{NT}_{i}}-\sum_{j=1}^{n} {G^{'}}_{{NT}_{j}}=\sum_{i=1}^{n} G_{{NT}_{i}}-\sum_{j=1}^{n} {G^{'}}_{{NT}_{j}}$$

$$X_{S}=\sum_{i=1}^{n} G_{T_{i}}-\sum_{j=1}^{n} {G^{'}}_{T_{j}} \begin{matrix} from \left( 1 \right) \\ = \end{matrix}\sum_{j=1}^{n} {G^{'}}_{{NT}_{j}}-\sum_{i=1}^{n} G_{{NT}_{i}}=-\left( \sum_{i=1}^{n} G_{{NT}_{i}}-\sum_{j=1}^{n} {G^{'}}_{{NT}_{j}} \right)$$

$$=-X_{(S)}$$

$$\therefore X_{T}+X_{S}=-(X_{\left( T \right)}+X_{\left( S \right)})$$

**(Case 2) The number of the affected sibs is greater than the number of the unaffected sibs. (*m*>*n*)**

In this case, $X_{T}$ and $X_{\left( T \right)}$ have the same form as in case 1. Therefore, we only discuss $X_{S}\mathrm{and}X_{\left( S \right)}$ here.

$$X_{S}=n\sum_{i=1}^{m} G_{T_{i}}-m\sum_{j=1}^{n} {G^{'}}_{T_{j}}$$

$$X_{(S)}=n\sum_{i=1}^{m} G_{{NT}_{i}}-m\sum_{j=1}^{n} {G^{'}}_{{NT}_{j}}$$

$$X_{S}= n\sum_{i=1}^{m} G_{T_{i}}-m\sum_{j=1}^{n} {G^{'}}_{T_{j}}$$

$$=n\sum_{i=1}^{n} G_{T_{i}}-n\sum_{j=1}^{n} {G^{'}}_{T_{j}}+n\sum_{i=n+1}^{m} G_{T_{i}}-\left( m-n \right)\sum_{j=1}^{n} {G^{'}}_{T_{j}}$$

$$= n\left( \sum_{i=1}^{n} G_{T_{i}}-\sum_{j=1}^{n} {G^{'}}_{T_{j}} \right)+\sum_{i=n+1}^{m} \left( G_{T_{i}}-{G^{'}}_{T_{1}} \right)+\ldots+\sum_{i=n+1}^{m} \left( G_{T_{i}}-{G^{'}}_{T_{n}} \right)$$

$$= n\left( \sum_{i=1}^{n} G_{T_{i}}-\sum_{j=1}^{n} {G^{'}}_{T_{j}} \right)+ \sum_{i=n+1}^{m} \sum_{j=1}^{n} (G_{T_{i}}-{G^{'}}_{T_{j}})$$

$$= n\left( \sum_{j=1}^{n} {G^{'}}_{{NT}_{j}}-\sum_{i=1}^{n} G_{{NT}_{i}} \right)+\sum_{i=n+1}^{m} \sum_{j=1}^{n} ({G^{'}}_{{NT}_{j}}-G_{{NT}_{i}})---according to (1)$$

$$X_{\left( S \right)}=n\sum_{i=1}^{m} G_{{NT}_{i}}-m\sum_{j=1}^{n} {G^{'}}_{{NT}_{j}}$$

$$= n\sum_{i=1}^{n} G_{{NT}_{i}}-n\sum_{j=1}^{n} {G^{'}}_{{NT}_{j}}+n\sum_{i=n+1}^{m} G_{{NT}_{i}}-\left( m-n \right)\sum_{j=1}^{n} {G^{'}}_{{NT}_{j}}$$

$$= n\left( \sum_{i=1}^{n} G_{{NT}_{i}}-\sum_{j=1}^{n} {G^{'}}_{{NT}_{j}} \right)+\sum_{i=n+1}^{m} \left( G_{{NT}_{i}}-{G^{'}}_{{NT}_{1}} \right)+\ldots+\sum_{i=n+1}^{m} \left( G_{{NT}_{i}}-{G^{'}}_{{NT}_{n}} \right)$$

$$= n\left( \sum_{i=1}^{n} G_{{NT}_{i}}-\sum_{j=1}^{n} {G^{'}}_{{NT}_{j}} \right)+ \sum_{i=n+1}^{m} \sum_{j=1}^{n} (G_{{NT}_{i}}-{G^{'}}_{{NT}_{j}})$$

$$X_{S}=n\left( \sum_{j=1}^{n} {G^{'}}_{{NT}_{j}}-\sum_{i=1}^{n} G_{{NT}_{i}} \right)+\sum_{i=n+1}^{m} \sum_{j=1}^{n} ({G^{'}}_{{NT}_{j}}-G_{{NT}_{i}})$$

$$= -\left\{ n\left( \sum_{i=1}^{n} G_{{NT}_{i}}-\sum_{j=1}^{n} {G^{'}}_{{NT}_{j}} \right)+ \sum_{i=n+1}^{m} \sum_{j=1}^{n} (G_{{NT}_{i}}-{G^{'}}_{{NT}_{j}}) \right\}=-X_{\left( S \right)}$$

$$\therefore X_{T}+X_{S}=-(X_{\left( T \right)}+X_{\left( S \right)})$$

**(Case 3) The number of the affected sibs is less than the number of the unaffected sibs. (*m*<*n*)**

In this case, $X_{T}$ and $X_{\left( T \right)}$ have the same form as in case 1. Therefore, we only discuss $X_{S}\mathrm{and}X_{\left( S \right)}$ here.

$$X_{S}= n\sum_{i=1}^{m} G_{T_{i}}-m\sum_{j=1}^{n} {G^{'}}_{T_{j}}$$

$$X_{(S)}=n\sum_{i=1}^{m} G_{{NT}_{i}}-m\sum_{j=1}^{n} {G^{'}}_{{NT}_{j}}$$

$$X_{S}=\sum_{i=1}^{m} G_{T_{i}}-m\sum_{j=1}^{n} {G^{'}}_{T_{j}}$$

$$= m\sum_{i=1}^{m} G_{T_{i}}-m\sum_{j=1}^{m} {G^{'}}_{T_{j}}+\left( n-m \right)\sum_{i=1}^{m} G_{T_{i}}-m\sum_{j=m+1}^{n} {G^{'}}_{T_{j}}$$

$$= m\left( \sum_{i=1}^{m} G_{T_{i}}-\sum_{j=1}^{m} {G^{'}}_{T_{j}} \right)+\sum_{i=1}^{m} \left( G_{T_{i}}-{G^{'}}_{T_{m+1}} \right)+\ldots+\sum_{i=1}^{m} \left( G_{T_{i}}-{G^{'}}_{T_{n}} \right)$$

$$= m\left( \sum_{i=1}^{m} G_{T_{i}}-\sum_{j=1}^{m} {G^{'}}_{T_{j}} \right)+ \sum_{i=1}^{m} \sum_{j=m+1}^{n} (G_{T_{i}}-{G^{'}}_{T_{j}})$$

$$= m\left( \sum_{j=1}^{m} {G^{'}}_{{NT}_{j}}-\sum_{i=1}^{m} G_{{NT}_{i}} \right)+\sum_{i=1}^{m} \sum_{j=m+1}^{n} ({G^{'}}_{{NT}_{j}}-G_{{NT}_{i}})--according to (2)$$

$$X_{\left( S \right)}=n\sum_{i=1}^{m} G_{{NT}_{i}}-m\sum_{j=1}^{n} {G^{'}}_{{NT}_{j}}$$

$$= m\sum_{i=1}^{m} G_{{NT}_{i}}-m\sum_{j=1}^{m} {G^{'}}_{{NT}_{j}}+(n-m)\sum_{i=1}^{m} G_{{NT}_{i}}-m\sum_{j=m+1}^{n} {G^{'}}_{{NT}_{j}}$$

$$= m\left( \sum_{i=1}^{m} G_{{NT}_{i}}-\sum_{j=1}^{m} {G^{'}}_{{NT}_{j}} \right)+\sum_{i=1}^{m} \left( G_{{NT}_{i}}-{G^{'}}_{{NT}_{m+1}} \right)+\ldots+\sum_{i=1}^{m} \left( G_{{NT}_{i}}-{G^{'}}_{{NT}_{n}} \right)$$

$$=m\left( \sum_{i=1}^{m} G_{{NT}_{i}}-\sum_{j=1}^{m} {G^{'}}_{{NT}_{j}} \right)+ \sum_{i=1}^{m} \sum_{j=m+1}^{n} (G_{{NT}_{i}}-{G^{'}}_{{NT}_{j}})$$

$$X_{S}= m\left( \sum_{j=1}^{m} {G^{'}}_{{NT}_{j}}-\sum_{i=1}^{m} G_{{NT}_{i}} \right)+\sum_{i=1}^{m} \sum_{j=m+1}^{n} ({G^{'}}_{{NT}_{j}}-G_{{NT}_{i}})$$

$$= -\left\{ m\left( \sum_{i=1}^{m} G_{{NT}_{i}}-\sum_{j=1}^{m} {G^{'}}_{{NT}_{j}} \right)+ \sum_{i=1}^{m} \sum_{j=m+1}^{n} (G_{{NT}_{i}}-{G^{'}}_{{NT}_{j}}) \right\}=-X_{\left( S \right)}$$

$$\therefore X_{T}+X_{S}=-(X_{\left( T \right)}+X_{\left( S \right)})$$

Because $\frac{1}{n_{T}+n_{S}}$ is a constant, $\frac{1}{n_{T}+n_{S}}\left( X_{T}+X_{S} \right)=-\frac{1}{n_{T}+n_{S}}\left( X_{(T)}+X_{(S)} \right)$ is satisfied in Cases 1,2and3.

Therefore, if we permute the transmitted and non-transmitted alleles simultaneously from both parents to all the children, it simply results in a sign change in the PDT statistic. The argument is still true when we have different numbers of affected and unaffected siblings.

Since there can be LD among SNPs, we need to permute haplotypes to preserve the LD structures among SNPs when analyzing multiple SNPs. We then discuss the relationship between the permutation of haplotypes and the single-SNP PDT statistics for two SNPs.

We use $\left( \begin{matrix} a \\ b \end{matrix} \right)$ to denote a haplotype for two SNPs, where *a* is the allele at the first SNP, and *b* is the allele at the second SNP.

Also consider a nuclear family with two parents, *m* affected and *n* unaffected siblings.

Let $H_{T_{i}}$ be the number of haplotype $\left( \begin{matrix} 1 \\ 1 \end{matrix} \right)$ transmitted from parents, and let $H_{{NT}_{i}}$ be the number of non-transmitted haplotype $\left( \begin{matrix} 1 \\ 1 \end{matrix} \right)$ from parents, to affected sib *i* , where *i*=1,2,…,*m*

Let ${H^{'}}_{T_{j}}$ be the number of haplotype $\left( \begin{matrix} 1 \\ 1 \end{matrix} \right)$ transmitted from parents, and let ${H^{'}}_{{NT}_{j}}$ be the number of non-transmitted haplotype $\left( \begin{matrix} 1 \\ 1 \end{matrix} \right)$ from parents, to unaffected sib *j* , where *j*=1,2,…,*n*

Table A.2 shows the transmitted and non-transmitted statistics for haplotype $\left( \begin{matrix} 1 \\ 1 \end{matrix} \right)$ for affected and unaffected sibs for the nuclear family.

Since the number of haplotype $\left( \begin{matrix} 1 \\ 1 \end{matrix} \right)$ in parents is fixed,$H_{T_{1}}+H_{{NT}_{1}}=H_{T_{2}}+H_{{NT}_{2}}=\ldots=H_{T_{m}}+H_{{NT}_{m}}={H^{'}}_{T_{1}}+{H^{'}}_{{NT}_{1}}={H^{'}}_{T_{2}}+{H^{'}}_{{NT}_{2}}=\ldots={H^{'}}_{T_{n}}+{H^{'}}_{{NT}_{n}}$

If *m*=*n*

$$H_{T_{1}}-{H^{'}}_{T_{1}}={H^{'}}_{{NT}_{1}}-H_{{NT}_{1}}$$

$$H_{T_{2}}-{H^{'}}_{T_{2}}={H^{'}}_{{NT}_{2}}-H_{{NT}_{2}}$$

.

.

.

$$H_{T_{n}}-{H^{'}}_{T_{n}}={H^{'}}_{{NT}_{n}}-H_{{NT}_{n}}$$

$$\therefore\sum_{i=1}^{n} H_{T_{i}}-\sum_{j=1}^{n} {H^{'}}_{T_{j}}=\sum_{j=1}^{n} {H^{'}}_{{NT}_{j}}-\sum_{i=1}^{n} H_{{NT}_{i}} -----(1)$$

$$\sum_{i=1}^{m} H_{T_{i}}-\sum_{j=1}^{m} {H^{'}}_{T_{j}}=\sum_{j=1}^{m} {H^{'}}_{{NT}_{j}}-\sum_{i=1}^{m} H_{{NT}_{i}} -----(2)$$

Let $X_{T}$ be the sum of the difference in the haplotype counts between the transmitted and the non-transmitted haplotypes overall all triads. Similarly, let $X_{S}$ be the sum of the difference in haplotype counts between the affected and unaffected sibs over all discordant sib pairs.

$$Therefore , X_{T}=\sum_{i=1}^{m} (H_{T_{i}}-H_{{NT}_{i}})$$

$$If m=n, X_{s}=\sum_{i=1}^{m} H_{T_{i}}-\sum_{j=1}^{n} {H^{'}}_{T_{j}}$$

$$If m\neq n, X_{s}=n\sum_{i=1}^{m} H_{T_{i}}-m\sum_{j=1}^{n} {H^{'}}_{T_{j}}$$

The PDT statistic for haplotype $\left( \begin{matrix} 1 \\ 1 \end{matrix} \right)$ for this nuclear family is calculated as $D_{\left( \begin{matrix} 1 \\ 1 \end{matrix} \right)}=\frac{1}{n_{T}+n_{S}}(X_{T}+X_{S})$

After permuting the transmitted and non-transmitted haplotypes, then

$$X_{(T)}=\sum_{i=1}^{m} (H_{{NT}_{i}}-H_{T_{i}})$$

$$X_{(S)}=\sum_{i=1}^{m} H_{{NT}_{i}}-\sum_{j=1}^{n} {H^{'}}_{{NT}_{j}}$$

$$P_{\left( \begin{matrix} 1 \\ 1 \end{matrix} \right)}=\frac{1}{n_{T}+n_{S}}(X_{(T)}+X_{(S)})$$

Using the similar procedures for the single-SNP situation, we can show that $D_{\left( \begin{matrix} 1 \\ 1 \end{matrix} \right)}=-P_{\left( \begin{matrix} 1 \\ 1 \end{matrix} \right)}$

The same procedure can also be applied to haplotypes $\left( \begin{matrix} 1 \\ 2 \end{matrix} \right),\left( \begin{matrix} 2 \\ 1 \end{matrix} \right),\left( \begin{matrix} 2 \\ 2 \end{matrix} \right)$ and the same conclusion can be made.

Define $D_{\left( \begin{matrix} a \\ b \end{matrix} \right)}$ is the PDT statistic for haplotype $\left( \begin{matrix} a \\ b \end{matrix} \right)$ at two SNPs

$P_{\left( \begin{matrix} a \\ b \end{matrix} \right)}$ is the PDT statistics for haplotype $\left( \begin{matrix} a \\ b \end{matrix} \right)$ after permuting the transmitted and non-transmitted haplotypes , where *a*=1,2 and *b*=1,2

Let the PDT statistics for allele 1 at the first and second SNPs be $D_{1}$ and $D_{1^{'}}$, respectively. Similarly, let the PDT statistics for allele 2 at the first and second SNPs be $D_{2}$ and $D_{2^{'}}$, respectively. It can be shown that

$$D_{1}=D_{\left( \begin{matrix} 1 \\ 1 \end{matrix} \right)}+D_{\left( \begin{matrix} 1 \\ 2 \end{matrix} \right)}=-P_{\left( \begin{matrix} 1 \\ 1 \end{matrix} \right)}-P_{\left( \begin{matrix} 1 \\ 2 \end{matrix} \right)}=-\left( P_{\left( \begin{matrix} 1 \\ 1 \end{matrix} \right)}+P_{\left( \begin{matrix} 1 \\ 2 \end{matrix} \right)} \right) =-D_{(1)}$$

$$D_{2}=D_{\left( \begin{matrix} 2 \\ 1 \end{matrix} \right)}+D_{\left( \begin{matrix} 2 \\ 2 \end{matrix} \right)}=-P_{\left( \begin{matrix} 2 \\ 1 \end{matrix} \right)}-P_{\left( \begin{matrix} 2 \\ 2 \end{matrix} \right)}=-\left( P_{\left( \begin{matrix} 2 \\ 1 \end{matrix} \right)}+P_{\left( \begin{matrix} 2 \\ 2 \end{matrix} \right)} \right) =-D_{(2)}$$

$$D_{1^{'}}=D_{\left( \begin{matrix} 1 \\ 1 \end{matrix} \right)}+D_{\left( \begin{matrix} 2 \\ 1 \end{matrix} \right)}=-P_{\left( \begin{matrix} 1 \\ 1 \end{matrix} \right)}-P_{\left( \begin{matrix} 2 \\ 1 \end{matrix} \right)}=-\left( P_{\left( \begin{matrix} 1 \\ 1 \end{matrix} \right)}+P_{\left( \begin{matrix} 2 \\ 1 \end{matrix} \right)} \right) =-D_{(1^{'})}$$

$$D_{2^{'}}=D_{\left( \begin{matrix} 1 \\ 2 \end{matrix} \right)}+D_{\left( \begin{matrix} 2 \\ 2 \end{matrix} \right)}=-P_{\left( \begin{matrix} 1 \\ 2 \end{matrix} \right)}-P_{\left( \begin{matrix} 2 \\ 2 \end{matrix} \right)}=-\left( P_{\left( \begin{matrix} 1 \\ 2 \end{matrix} \right)}+P_{\left( \begin{matrix} 2 \\ 2 \end{matrix} \right)} \right) =-D_{(2^{'})}$$

Thus, if we permute the transmitted and non-transmitted haplotypes, it simply results in simultaneous sign changes in the PDT statistics for the two SNPs.

We then check if the results are still true when a haplotype has recombination.

Assume we consider a nuclear family with one affected and one unaffected siblings and only the affected sib has a haplotype with recombination from the mother.

We only consider the case where the genotypes at the two SNPs in the mother are both heterozygous. If at least one SNP is homozygous, the observation of haplotype in the affected sib is the same as the situation of no recombination. Then the same proof from the condition of no recombination can be applied.

Therefore, we consider the condition that the mother has haplotype $\left( \begin{matrix} 1 \\ 1 \end{matrix} \right)\left( \begin{aligned} 2 \\ 2 \end{aligned} \right)$. Then, we would like to show that $D_{\left( \begin{matrix} 1 \\ 1 \end{matrix} \right)}+D_{\left( \begin{matrix} 1 \\ 2 \end{matrix} \right)}=-P_{\left( \begin{matrix} 1 \\ 1 \end{matrix} \right)}-P_{\left( \begin{matrix} 1 \\ 2 \end{matrix} \right)}$ is still true.

Let $T_{A}^{ab}$ be the number of haplotype $\left( \begin{matrix} a \\ b \end{matrix} \right)$ transmitted from parents and ${NT}_{A}^{ab}$ be the number of non-transmitted haplotype $\left( \begin{matrix} a \\ b \end{matrix} \right)$ from parents to the affected child.

Let $T_{U}^{ab}$ be the number of haplotype $\left( \begin{matrix} a \\ b \end{matrix} \right)$ transmitted from parents and ${NT}_{U}^{ab}$ be the number of non-transmitted haplotype $\left( \begin{matrix} a \\ b \end{matrix} \right)$ from parents, to the unaffected child.

Table A.3 summarizes the transmitted and non-transmitted statistics for haplotype $\left( \begin{matrix} a \\ b \end{matrix} \right)$ for the affected and unaffected siblings.

For haplotype $\left( \begin{matrix} 1 \\ 1 \end{matrix} \right)$

If there is no recombination in the mother, then the number of haplotype $\left( \begin{matrix} 1 \\ 1 \end{matrix} \right)$ in parents is fixed, $T_{A}^{11}+{NT}_{A}^{11}=T_{U}^{11}+{NT}_{U}^{11}$

However, since haplotype $\left( \begin{matrix} 1 \\ 1 \end{matrix} \right)$ has recombination in the mother and only the affected child has the haplotype with recombination from the mother, the number of haplotype $\left( \begin{matrix} 1 \\ 1 \end{matrix} \right)$ in the affected child is the number of haplotype $\left( \begin{matrix} 1 \\ 1 \end{matrix} \right)$ in the unaffected child minus 1. Therefore, $T_{A}^{11}+{NT}_{A}^{11}+1=T_{U}^{11}+{NT}_{U}^{11}$

$\therefore T_{A}^{11}-T_{U}^{11}={NT}_{U}^{11}-{NT}_{A}^{11}-1$ $-----(1)$

Let $X_{T_{\left( \begin{matrix} 1 \\ 1 \end{matrix} \right)}}$ be the difference in the haplotype counts between the transmitted and non-transmitted haplotypes. Similarly, let $X_{S_{\left( \begin{matrix} 1 \\ 1 \end{matrix} \right)}}$ be the difference in the haplotype counts between the affected and the unaffected siblings.

$$Therefore ,X_{T_{\left( \begin{matrix} 1 \\ 1 \end{matrix} \right)}}=T_{A}^{11}-{NT}_{A}^{11} , X_{S_{\left( \begin{matrix} 1 \\ 1 \end{matrix} \right)}}= T_{A}^{11}-T_{U}^{11}$$

In this case, $n_{T}=$1 and $n_{S}=$1

The PDT statistic for haplotype $\left( \begin{matrix} 1 \\ 1 \end{matrix} \right)$ for this nuclear family is calculated as $D_{\left( \begin{matrix} 1 \\ 1 \end{matrix} \right)}=\frac{1}{2}(X_{T_{\left( \begin{matrix} 1 \\ 1 \end{matrix} \right)}}+X_{S_{\left( \begin{matrix} 1 \\ 1 \end{matrix} \right)}})$

After permuting the transmitted and non-transmitted labels, then

$$P_{T_{\left( \begin{matrix} 1 \\ 1 \end{matrix} \right)}}={NT}_{A}^{11}-T_{A}^{11}$$

$$P_{S_{\left( \begin{matrix} 1 \\ 1 \end{matrix} \right)}}={NT}_{A}^{11}-{NT}_{U}^{11}$$

Therefore, $X_{T_{\left( \begin{matrix} 1 \\ 1 \end{matrix} \right)}}=T_{A}^{11}-{NT}_{A}^{11}=-\left( {NT}_{A}^{11}-T_{A}^{11} \right)=-P_{T_{\left( \begin{matrix} 1 \\ 1 \end{matrix} \right)}}$

$X_{S_{\left( \begin{matrix} 1 \\ 1 \end{matrix} \right)}}= T_{A}^{11}-T_{U}^{11}={NT}_{U}^{11}-{NT}_{A}^{11}-1=-P_{S_{\left( \begin{matrix} 1 \\ 1 \end{matrix} \right)}}-1 ---by (1)$

For haplotype $\left( \begin{matrix} 1 \\ 2 \end{matrix} \right)$

Similar to haplotype $\left( \begin{matrix} 1 \\ 1 \end{matrix} \right)$, we can show that $T_{A}^{12}+{NT}_{A}^{12}=T_{U}^{12}+{NT}_{U}^{12}+1$

$\therefore T_{A}^{12}-T_{U}^{12}={NT}_{U}^{12}-{NT}_{A}^{12}+1$ $-----(2)$

$$Also define X_{T_{\left( \begin{matrix} 1 \\ 2 \end{matrix} \right)}}=T_{A}^{12}-{NT}_{A}^{12} , X_{S_{\left( \begin{matrix} 1 \\ 2 \end{matrix} \right)}}= T_{A}^{12}-T_{U}^{12}$$

The PDT statistic for haplotype $\left( \begin{matrix} 1 \\ 2 \end{matrix} \right)$ for this nuclear family is calculated as $D_{\left( \begin{matrix} 1 \\ 2 \end{matrix} \right)}=\frac{1}{2}(X_{T_{\left( \begin{matrix} 1 \\ 2 \end{matrix} \right)}}+X_{S_{\left( \begin{matrix} 1 \\ 2 \end{matrix} \right)}})$

After permuting the transmitted and non-transmitted haplotypes, then

$$P_{T_{\left( \begin{matrix} 1 \\ 2 \end{matrix} \right)}}={NT}_{A}^{12}-T_{A}^{12}$$

$$P_{S_{\left( \begin{matrix} 1 \\ 2 \end{matrix} \right)}}={NT}_{A}^{12}-{NT}_{U}^{12}$$

Therefore, $X_{T_{\left( \begin{matrix} 1 \\ 2 \end{matrix} \right)}}=T_{A}^{12}-{NT}_{A}^{12}=-\left( {NT}_{A}^{12}-T_{A}^{12} \right)=-P_{T_{\left( \begin{matrix} 1 \\ 2 \end{matrix} \right)}}$

$X_{S_{\left( \begin{matrix} 1 \\ 2 \end{matrix} \right)}}= T_{A}^{12}-T_{U}^{12}={NT}_{U}^{12}-{NT}_{A}^{12}+1=1-P_{S_{\left( \begin{matrix} 1 \\ 2 \end{matrix} \right)}} ---by (2)$

$$\therefore D_{\left( \begin{matrix} 1 \\ 1 \end{matrix} \right)}+D_{\left( \begin{matrix} 1 \\ 2 \end{matrix} \right)}=\frac{1}{n_{T}+n_{S}}\left( X_{T_{\left( \begin{matrix} 1 \\ 1 \end{matrix} \right)}}+X_{S_{\left( \begin{matrix} 1 \\ 1 \end{matrix} \right)}}+X_{T_{\left( \begin{matrix} 1 \\ 2 \end{matrix} \right)}}+X_{S_{\left( \begin{matrix} 1 \\ 2 \end{matrix} \right)}} \right)=\frac{1}{n_{T}+n_{S}}\left( -P_{T_{\left( \begin{matrix} 1 \\ 1 \end{matrix} \right)}}-P_{S_{\left( \begin{matrix} 1 \\ 1 \end{matrix} \right)}}-1 -P_{T_{\left( \begin{matrix} 1 \\ 2 \end{matrix} \right)}}+1-P_{S_{\left( \begin{matrix} 1 \\ 2 \end{matrix} \right)}} \right)=-\frac{1}{n_{T}+n_{S}}\left( P_{T_{\left( \begin{matrix} 1 \\ 1 \end{matrix} \right)}}+P_{S_{\left( \begin{matrix} 1 \\ 1 \end{matrix} \right)}}+P_{T_{\left( \begin{matrix} 1 \\ 2 \end{matrix} \right)}}+P_{S_{\left( \begin{matrix} 1 \\ 2 \end{matrix} \right)}} \right) =-P_{\left( \begin{matrix} 1 \\ 1 \end{matrix} \right)}-P_{\left( \begin{matrix} 1 \\ 2 \end{matrix} \right)}$$

The argument is still true when we have different numbers of affected and unaffected siblings. The procedure can also be extended to multiple SNPs and it can be shown that permuting transmitted and non-transmitted haplotypes at multiple SNPs results in simultaneous sign changes for all of the SNPs.

**Figures**


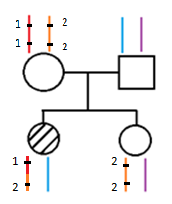


Figure 1.A. Independent haplotypes are labeled with different colors. Haplotypes transmitted from the mother to the affected sib have a crossover between the two SNPs.

**Tables**

Table A.1

|  | Affected  sib 1 | Affected  sib 2 |  | Affected  sib *m* | Unaffected  sib 1 | Unaffected  sib 2 |  | Unaffected  sib *n* |
| --- | --- | --- | --- | --- | --- | --- | --- | --- |
| Transmitted | $G_{T_{1}}$ | $G_{T_{2}}$ | … | $G_{T_{m}}$ | ${G^{'}}_{T_{1}}$ | ${G^{'}}_{T_{2}}$ | … | ${G^{'}}_{T_{n}}$ |
| Non  Transmitted | $G_{{NT}_{1}}$ | $G_{{NT}_{2}}$ | … | $G_{{NT}_{m}}$ | ${G^{'}}_{{NT}_{1}}$ | ${G^{'}}_{{NT}_{2}}$ | … | ${G^{'}}_{{NT}_{n}}$ |

Table A.2

|  | Affected | Affected |  | Affected | Unaffected | Unaffected |  | Unaffected |
| --- | --- | --- | --- | --- | --- | --- | --- | --- |
| Transmitted | $H_{T_{1}}$ | $H_{T_{2}}$ | … | $H_{T_{m}}$ | ${H^{'}}_{T_{1}}$ | ${H^{'}}_{T_{2}}$ | … | ${H^{'}}_{T_{n}}$ |
| Non  Transmitted | $H_{{NT}_{1}}$ | $H_{{NT}_{2}}$ | … | $H_{{NT}_{m}}$ | ${H^{'}}_{{NT}_{1}}$ | ${H^{'}}_{{NT}_{2}}$ | … | ${H^{'}}_{{NT}_{n}}$ |

Table A.3

|  | Affected | Unaffected |
| --- | --- | --- |
| Transmitted | $T_{A}^{ab}$ | $T_{U}^{ab}$ |
| Non Transmitted | ${NT}_{A}^{ab}$ | ${NT}_{U}^{ab}$ |
